# Gene flow and the genealogical history of *Heliconius heurippa*

**DOI:** 10.1186/1471-2148-8-132

**Published:** 2008-05-02

**Authors:** Camilo Salazar, Chris D Jiggins, Jesse E Taylor, Marcus R Kronforst, Mauricio Linares

**Affiliations:** 1 Instituto de Genética, Departamento de Ciencias Biologicas, Universidad de los Andes, P.O. Box 4976, Bogotá, Colombia; 2 University of Cambridge, Department of Zoology, Downing street, Cambridge CB2 3EJ, UK; 3 Department of Statistics, Oxford University, 1 South Parks Road, Oxford, OX1 3TG, UK; 4 FAS, Center for Systems Biology, Harvard University, 7 Divinity Avenue Cambridge, MA 02138, USA

## Abstract

**Background:**

The neotropical butterfly *Heliconius heurippa *has a hybrid colour pattern, which also contributes to reproductive isolation, making it a likely example of hybrid speciation. Here we used phylogenetic and coalescent-based analyses of multilocus sequence data to investigate the origin of *H. heurippa*.

**Results:**

We sequenced a mitochondrial region (CoI and CoII), a sex-linked locus (*Tpi*) and two autosomal loci (*w *and *sd*) from *H. heurippa *and the putative parental species, *H. cydno *and *H. melpomene*. These were analysed in combination with data from two previously sequenced autosomal loci, *Dll *and *Inv*. *H. heurippa *was monophyletic at mtDNA and *Tpi*, but showed a shared distribution of alleles derived from both parental lineages at all four autosomal loci. Estimates of genetic differentiation showed that *H. heurippa *is closer to *H. cydno *at mtDNA and three autosomal loci, intermediate at *Tpi*, and closer to *H. melpomene *at *Dll*. Using coalescent simulations with the Isolation-Migration model (IM), we attempted to establish the incidence of gene flow in the origin of *H. heurippa*. This analysis suggested that ongoing introgression is frequent between all three species and variable in extent between loci.

**Conclusion:**

Introgression, which is a necessary precursor of hybrid speciation, seems to have also blurred the coalescent history of these species. The origin of *Heliconius heurippa* may have been restricted to introgression of few colour pattern genes from *H. melpomene* into the *H. cydno* genome, with little evidence of genomic mosaicism.

## Background

Homoploid hybrid speciation, in which a hybrid lineage becomes established as a novel species without a change in chromosome number, is thought to be rare and has only been well documented in a handful of plant and animals species [1-7]. Indeed, most of what is known is based on detailed studies of just one group, the *Helianthus *sunflowers. Notably, the species *Helianthus anomalus *has a clearly hybrid genome in which large blocks of the genome are derived from one or other of the parental species, *Helianthus petiolaris *and *Helianthus annus *[8]. The most convincing evidence comes from the fact that synthetic hybrids between the two species show a similar genomic composition and some ecological characteristics of the natural hybrid lineage [9]. This work has led to a view of homoploid hybrid speciation in which the derived hybrid lineage has a genome comprising similar proportions of the two parental genomes [4].

Nonetheless, this is not the only way in which hybridization might contribute to speciation. In cichlid fishes and Darwin's finches for example, it has been suggested that adaptive radiation might have been facilitated by evolutionary novelty generated through hybridization [10,11]. There is also strong evidence for gene flow at adaptive loci in the Hawaiian silverswords and in smelt fishes [12,13]. Thus, novel traits that directly affect adaptive divergence and/or reproductive isolation might become established as a result of introgression [14,15]. If the traits also have pleiotropic effects on reproductive isolation (i.e. 'magic traits' sensu Gavrilets 2004 [16]), hybridization could make a direct contribution to reproductive isolation of a novel lineage and hence, to speciation [5,17]. Under this scenario, if establishment of the hybrid trait involved many generations of backcrossing, then the genome of the novel hybrid linage could be predominantly derived from one of the parental species [17]. Regions of the genome not under selection might also be subject to gene flow through occasional ongoing hybridization between hybrid and parental species [18,19]. In *Heliconius *butterflies, Müllerian mimicry is common, often between related but non-sister species [20,21]. This implies repeated parallel evolution of shared colour patterns from an ancestral phenotype [20]. However, an alternative is that similar colour elements might be homologous and transferred between species through occasional hybridization and backcrossing events [20]. Natural hybrids show that 27% of species have inter-specific hybridization and that backcrosses are fairly common [20]. Thus, *Heliconius *pattern diversity could be facilitated by the movement of pre-established colour pattern adaptations [20,22,23].

*Heliconius heurippa *appears to have speciated via the hybrid origin of its novel colour pattern, which shares elements derived from both *H. melpomene *and *H. cydno *[23-26]. When *H. c. cordula *and *H. m. melpomene*, subspecies that occur near the current range of *H. heurippa *on the eastern slope of the Colombian Andes are crossed, a virtually identical colour pattern to that of *H. heurippa *can be created in the laboratory after just three generations [26]. Crosses between *H. heurippa *and these artificial hybrids show that the pattern breeds true implying genetic homology between the different forms, although this remains to be proven at a molecular genetic level [26]. *Heliconius *colour patterns are aposematic and often mimetic, such that rare colour pattern hybrids are selected against by predators [27,28]. Thus, divergent colour pattern probably contributes to post-mating isolation between the species. In addition, behavioural experiments show that the combined hybrid colour pattern of *H. heurippa *is critical for mate recognition [26]. Removal of either the red element derived from *H. melpomene*, or the yellow element derived from *H. cydno*, results in the pattern being less attractive to *H. heurippa *males. These data therefore provide strong evidence that the *Heliconius heurippa *colour pattern is a hybrid trait that causes reproductive isolation.

Nonetheless, it remains unclear whether *H. heurippa *arose via a hybrid founding of the genome, similar to *Helianthus anomalus*, or through introgression of a few *H. melpomene *colour pattern alleles into the genome of *H. cydno*. In between these extremes, a variety of intermediate scenarios could be envisaged with varying levels of ongoing gene flow during speciation. Ecological and genetic studies indicate that *H. heurippa *is most closely related to *H. cydno*. Ecologically, *H. heurippa *is a geographic replacement of *H. cydno*, with similar habitat and altitudinal preferences. Crosses show female hybrid sterility between *H. heurippa *and *H. melpomene*, but complete compatibility between *H. heurippa *and *H. cydno *[24]. However, microsatellite data show that *H. heurippa *is a distinct species and not simply a geographic race of *H. cydno*; *H. heurippa *is considerably more differentiated than any other geographic populations of *H. melpomene *or *H. cydno *sampled in Panama, Colombia and Venezuela [26]. Additionally, *H. heurippa *had an intriguing pattern at two nuclear loci (*invected *and *Distal-less*). In both *H. heurippa *shares haplotypes with *H. melpomene *and *H. cydno *[26,29]. Here we examine the incidence of gene flow in the speciation history of *H. heurippa*, using sequences of these genes and fragments of five additional genes.

## Results

### Description of gene regions

We sequenced a mitochondrial region of 1572 bp representing 799 bp of CoI, 62 bp of the tRNA^LEU ^and 711 bp of CoII from 11 individuals. Including sequences from GenBank, the alignment had 1572 nucleotide sites examined from 69 individuals, of which 155 (10%) were variable. We obtained 584 bp of the Z-linked Triose phosphate isomerase exon 3 (31 bp), intron 3 (430 bp) and exon 4 (123 bp), for 11 alleles from 11 individuals. Including sequences from GenBank the total alignment had 25 alleles from 22 individuals. Identity was confirmed by comparison with reference sequences for *H. c*. *chioneus *(AF413788) and *H. m. rosina *(AF413790).

The four autosomal regions were portions of nuclear loci initially chosen for their potential role in the development of butterfly wing patterns [30]. Three of these, *Distal-less*, *invected *and *scalloped*, are transcription factors [31-33], while the fourth, *white*, is member of the ommochrome biosynthesis pathway that generates the yellow, orange and red pigments in *Heliconius *[34]. However, linkage mapping has shown that none of these genes are linked to the switch genes that control pattern divergence in *Heliconius *[35,36], so there is currently no evidence that they are involved in pattern evolution. We included 558 bp of *Distal-less*, corresponding to exon 4/5 (175 bp), intron 5 (333 bp) and exon 6 (50 bp) of *Drosophila *(NM166689- intron 4 is absent in *Heliconius*) for 32 alleles from 20 individuals; 439 bp of *invected *exon 2 (67 bp), intron 2 (265 bp) and exon 3 (107 bp) for 42 alleles from 23 individuals. We obtained 499 bp of *white *exon 4 (49 bp), intron 4 (397 bp) and exon 5 (53 bp) for 25 alleles from 17 individuals; 483 bp of the *scalloped *gene from exon 7 (13 bp), intron 7 (334 bp) and exon 8 (136 bp) for 22 alleles from 12 individuals.

### Species relationships and population genetic parameters

**Figure 1:**
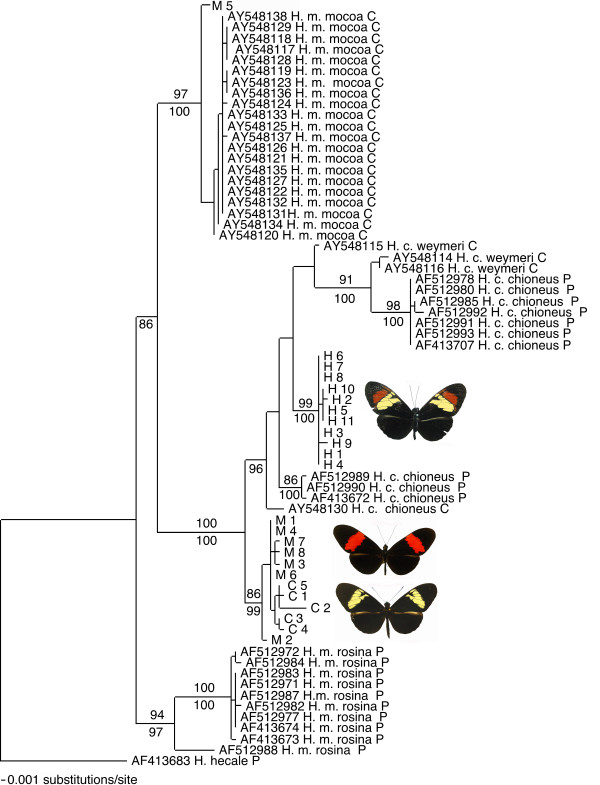
**mtDNA Phylogenetic tree**. Phylogenetic relationships of *H. heurippa *(H) with other populations of *H. melpomene *and *H. cydno *based on CoI and CoII sequences. C and M identify *H. m. melpomene *and *H. c. cordula *individuals of the putative parental species. Sequence ID's beginning with AF and AY indicate GenBank accession numbers. Branch lengths and probability values (under branches) were estimated using Bayesian analysis and bootstrap support (over branches) derived from a Maximum Parsimony analysis. Countries of origin are identified using the following abbreviations: P = Panama and C = Colombia; Abbreviations of species names are m. = *melpomene*, c. = *cydno*.

None of the three species formed a monophyletic group at all of the genes sampled. In the phylogeny derived from mitochondrial DNA, individuals of the three species fell into three well-supported monophyletic clades (Figure 1): 1) an eastern *melpomene *clade; 2) the *cydno *clade including all the *H. cydno *and *H. heurippa *sampled and seven *H. m. melpomene *from the eastern Andean foothills; 3) a western *melpomene *clade. Within the *cydno *clade, the *H. heurippa *haplotypes form a monophyletic group with five fixed differences from *H. cydno *(0.92% net divergence). Genetic diversity in *H. heurippa *was the lowest of the three species (Table 1). Tajima's D was not significantly different from zero (Table 1), suggesting that a small but constant population size rather than a recent bottleneck is more likely to explain the lack of variation in *H. heurippa*. Interestingly Tajima's D estimates were significantly negative for two of the three populations of *H. melpomene *(*H. m. rosina *and *H. m. mocoa*) included here for comparison (Table 1), possibly reflecting a recent bottleneck or mtDNA selective sweep.

**Table 1:** Summary of genetic polymorphism data for mtDNA sequences in each population

Population	N^a^	S^b^	θ_W_^c^	θ_W _95% CI^d^	D_T_^e^	D_T _|null
*H. m. mocoa*	22	9	0.0016	0.0014–0.0018	-1.8296	< 0.05*
*H. c. chioneus*	10	28	0.0064	0.0020–0.0108	0.3504	>0.1
*H. heurippa*	11	4	0.0009	0.0001–0.0017	-0.83418	>0.1
*H. m. melpomene*	8	39	0.0099	0.0045–0.0152	-1.5379	>0.05
*H. c. cordula*	5	9	0.0028	0.0025–0.0031	-0.1974	>0.1
*H. m. rosina*	10	26	0.0060	0.0037–0.0083	-1.8174	< 0.05*

*H. c. weymeri *and *H. c. chioneus *sequences from Colombia were excluded in the polymorphism analysis, because of the low number individuals available in each case (3 and 1, respectively). * Indicates statistical significance for departure of D_T _from neutral expectation at 95%. ^a ^Number of alleles. ^b ^Segregating sites. ^c ^Genetic diversity. ^d ^Genetic diversity 95% confidence interval. ^e ^Tajima's parameter.

**Figure 2:**
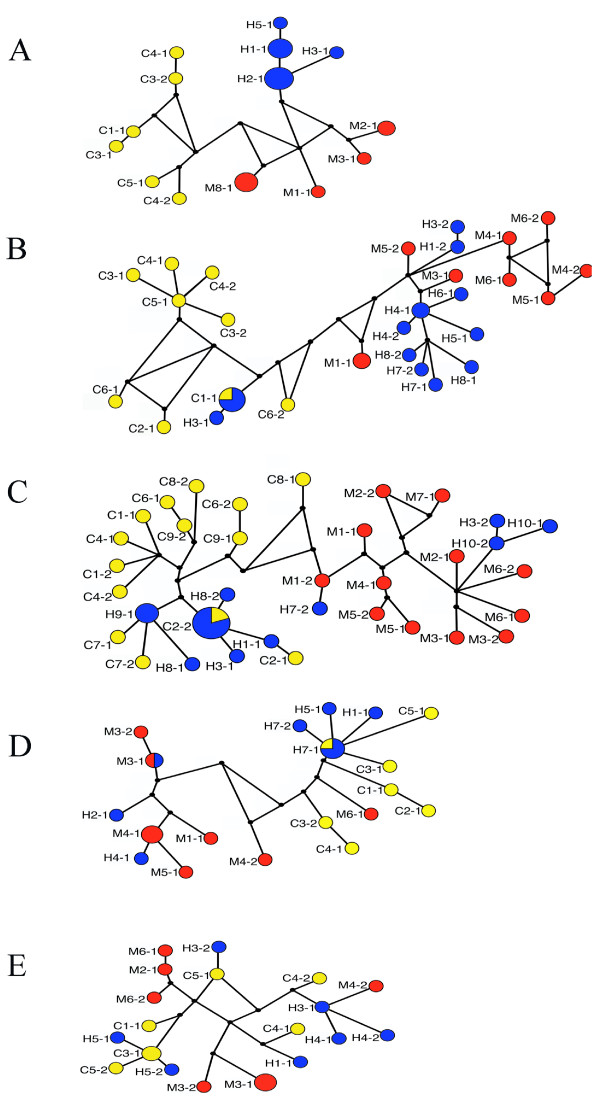
**Allele networks for nuclear genes**. Yellow, Red and Blue are *H. c. cordula*, *H. m. melpomene *and *H. heurippa *alleles. Respective alleles are also identified with the letters C, M and H, followed by the individual number and allele number. Black dots are hypothetical ancestors. Sizes of the circles reflect allele frequencies in the population. (A) *Tpi*, (B) *Dll*, (C)*inv*, (D) *w *and (E) *sd*.

Among the five nuclear loci, the sex-linked locus *Tpi *was the only marker that clearly separated all three species. *H. c. cordula *and *H. m. melopomene *alleles formed distinct clades separated by five fixed differences and one shared polymorphism, with 1.3% net divergence (Figure 2A). *H. heurippa *alleles also formed a distinct cluster (Figure 2A) separated by five fixed differences from *H. m. melpomene *(1.3% net divergence) and by six from *H. c. cordula *(1.4% net divergence). In concordance with the network groups, F_ST _values showed the species as three distinct populations, with *H. heurippa *showing greater differentiation from *H. c. cordula *(F_ST _= 0.791) and *H. m. melpomene *(F_ST _= 0.719) than that observed between *H. c. cordula *and *H. m. melopomene *(F_ST _= 0.498 P < 0.05; Table 3; Figure 2A).

Three of the autosomal loci, *Dll*, *inv *and *w*, show a striking pattern in which *H. c. cordula *and *H. m. melpomene *are clearly differentiated, but *H. heurippa *shares variation with both species. At *Dll, H. c. cordula *and *H. m. melpomene *(Figure 2B) are separated by 10 fixed differences (3% net divergence) and share three polymorphisms. Similarly at *inv*, *H. c. cordula *and *H. m. melpomene *have ten fixed differences (4.6% net divergence) and two shared polymorphisms (Figure 2C). At *w*, one allele of *H. m. melpomene *was shared with *H. c. cordula *(M6-1; Figure 2D), such that there was only one fixed difference (2% net divergence) and eight shared polymorphisms. Nonetheless, estimates of F_ST _between *H. c. cordula *and *H. m. melpomene *were high for all three loci (*Dll*, 0.621, *inv*, 0.593 and *w*, 0.327). In contrast, *H. heurippa *did not have any fixed differences with either *H. c. cordula *or *H. m. melpomene *at any of these three loci. At *Dll, H. heurippa *was more similar to *H. m. melpomene *(net divergence 0.41%) than *H. c. cordula *(net divergence 2.28%). At the other two loci, *H. heurippa *was more similar to *H. cydno*, with *inv *showing 0.56% and 2.76% divergence with *H. c. cordula *and *H. m. melpomene *respectively, while *w *showed 0.024% and 1% for the same two comparisons. Estimates of F_ST _supported these observations, with *Dll *showing *H. heurippa *not significantly differentiated from *H. m. melpomene *but strongly differentiated from *H. c. cordula *(Table 3), while *inv *and *w *showed the opposite pattern with *H. heurippa *more similar to *H. c. cordula *(Table 3).

In contrast with the patterns observed in the other five loci, the *sd *locus showed no fixed differences among any of the three species (Figure 2E). *H. c. cordula *and *H. m. melpomene *had seven shared polymorphisms and net divergence of 0.62%. *H. heurippa *shared 14 and ten polymorphisms with *H. c. cordula *and *H. m. melpomene *respectively, representing net divergence of 0.10% and 0.5%. Genetic diversity was generally high in all three species (Table 2). Tajima's D was significantly negative in *H. c. cordula *(Table 2). F_ST _values showed that *H. heurippa *was more similar to *H. c. cordula *than to *H. m. melpomene *(Table 3).

**Table 2:** Summary of genetic polymorphism data for nuclear sequences, in *H. c. cordula, H. heurippa and H. m. melpomene *populations.

*H. c. cordula*									
locus	n^a^	S^b^	θ_W_^c^	θ_W _95% CI^d^	D_T_^e^	D_T _95% CI^f^	P	D_T _rec 95% CI^g^	P
*Tpi*	6	9	0.0083	0.0037–0.0129	-0.1548	(-1.4347 to1.5822)	0.4850	(-0.8862 to 0.9428)	0.4150
*Dll*	9	23	0.0193	0.0101–0.0285	-0.5311	(-1.6696 to1.6159)	0.3300	(-0.7913 to 0.7376)	0.0760
*inv*	14	51	0.0394	0.0177–0.0571	-1.1859	(-1.6520 to1.6498)	0.1000	(-0.6055 to 0.5822)	0.000001*
W	7	26	0.0281	0.0064–0.0498	-0.3275	(-1.5502 to1.5575)	0.4070	(-0.6332 to 0.6404)	0.1710
*Sd*	7	20	0.0171	0.0128–0.0213	-0.8328	(-1.5533 to1.7872)	0.2440	(-0.7018 to 0.8047)	0.0090*

*H. heurippa*									
locus	n^a^	S^b^	θ_W_^c^	θ_W _95% CI^d^	D_T_^e^	D_T _95% CI^f^	P	D_T _rec 95% CI^g^	P

*Tpi*	11	2	0.0014	0.0008–0.0036	0.199	(-1.4296 to1.8276)	0.5920	(-1.4296 to 1.6648)	0.5320
*Dll*	14	29	0.0229	0.0126–0.0331	-0.642	(-1.7616 to1.6671)	0.2950	(-0.7520 to 0.6874)	0.0420
*inv*	16	42	0.0307	0.0198–0.0416	0.0026	(-1.8007 to1.7247)	0.5590	(-0.6635 to 0.6064)	0.5060
W	9	29	0.0340	0.0246–0.0434	0.1089	(-1.6834 to1.5982)	0.5850	(-0.6231 to 0.6390)	0.6450
*Sd*	7	25	0.0215	0.0127–0.0302	-0.0604	(-1.5947 to1.6325)	0.5260	(-0.6953 to 0.62731)	0.4530

*H. m. melpomene*									
locus	n^a^	S^b^	θ_W_^c^	θ_W _95% CI^d^	D_T_^e^	D_T _95% CI^f^	P	D_T _rec 95% CI^g^	P

*Tpi*	8	9	0.0078	0.0043–0.0113	1.2504	(-1.6740 to1.7414)	0.9120	(-1.0680 to 0.9984)	0.9890
*Dll*	9	21	0.0169	0.0048–0.0217	-0.6685	(-1.6864 to1.5708)	0.3010	(-0.7703 to 0.7226)	0.0510
*inv*	12	29	0.0235	0.0170–0.0300	-1.7198	(-1.7129 to1.6628)	0.024*	(-0.6769 to 0.6816)	0.000001*
W	9	31	0.0377	0.0341–0.0413	-1.1847	(-1.6591 to1.6624)	0.1130	(-0.6389 to 0.6184)	0.000001*
*Sd*	8	33	0.0266	0.0161–0.0371	-0.3359	(-1.5973 to1.6506)	0.3790	(-0.6258 to 0.5921)	0.1320

* Indicates a P=Pr(D≤ observed value) statistically significant deviation of Tajima's D from Hudson 1990 panmixia model [72]. 95% CIs under the model were calculated by coalescent simulations (with fixed S). The two-tailed probability test of significance was derived from simulations (P = 0.025). ^a ^Number of alleles. ^b ^Segregating sites. ^c ^Genetic diversity. ^d ^Genetic diversity 95% confidence interval. ^e ^Tajima's D. ^f ^Tajima's D, 95% confidence interval calculated without recombination. ^g ^Tajima's D, 95% confidence interval calculated with recombination.

**Table 3:** Genetic structure (F_ST_) values for comparisons between the three populations

mtDNA			
Population	*H. c. cordula*	*H. heurippa*	*H. m. melpomene*
*H. c. cordula *(5)	-		
*H. heurippa *(11)	0.905		
	0.0001*	-	
*H. m. melpomene *(8)	0.058	0.739	-
	0.44	0.0001*	

*Tpi*			
Population	*H. c. cordula*	*H. heurippa*	*H. m. melpomene*

*H. c. cordula *(6)	-		
*H. heurippa *(11)	0.791		
	0.0001*	-	
*H. m. melpomene *(8)	0.498	0.719	-
	0.0001*	0.0001*	

*Dll*			
Population	*H. c. cordula*	*H. heurippa*	*H. m. melpomene*

*H. c. cordula *(9)	-		
*H. heurippa *(14)	0.479		
	0.0001*	-	
*H. m. melpomene *(9)	0.621	0.085	-
	0.0001*	0.066	

*Inv*			
Population	*H. c. cordula*	*H. heurippa*	*H. m. melpomene*

*H. c. cordula *(14)	-		
*H. heurippa *(16)	0.107		
	0.022*	-	
*H. m. melpomene *(12)	0.593	0.419	-
	0.0001*	0.0001*	

*W*			
Population	*H. c. cordula*	*H. heurippa*	*H. m. melpomene*

*H. c. cordula *(7)	-		
*H. heurippa *(9)	0.004		
	0.383	-	
*H. m. melpomene *(9)	0.327	0.176	-
	0.003*	0.019*	

*Sd*			
Population	*H. c. cordula*	*H. heurippa*	*H. m. melpomene*

*H. c. cordula *(7)	-		
*H. heurippa *(7)	0.037	-	
	0.196		
*H. m. melpomene *(8)	0.148	0.119	-
	0.006*	0.020*	

* Significance at 95% was obtained by bootstrapping (10000 subsamples).

### Recombination analysis and IM input files

In order to select the 'basic' dataset required by the IM software, indel-free alignments were investigated to search for evidence of recombination for each species pair comparison [37]. In the *H. cydno *– *H. melpomene *comparison, recombination was only detected at *Dll *with its maximum significant breakpoint found between sequences C6-2 and M5-2 (p = 0.007) between 16^th ^and the 17^th ^sites, as was indicated using the maximum chi-square method. A recombination free block of 206 bp was selected in the 5' to 3' direction from the site of probable recombination (Table 4).

**Table 4:** IM blocks and mutation rate estimates for each species comparison.

				95% credibility interval	
Comparison		L (pb)	Estimate	Lower Limit	Upper Limit
*H. cydno-H. melpomene*	μ_*Co*_	1517	2.88 × 10^-9^	1.7 × 10^-9^	6.6 × 10^-9^
	μ_*Tpi*_	416	0.642 × 10^-9^	0.377 × 10^-9^	1.3 × 10^-9^
	μ_*Dll*_	206	0.491 × 10^-9^	0.282 × 10^-9^	0.868 × 10^-9^
	μ_*inv*_	391	1.6 × 10^-9^	0.792 × 10^-9^	2.3 × 10^-9^
	μ_*sd*_	473	1.1 × 10^-9^	0.739 × 10^-9^	1.9 × 10^-9^
	μ_*W*_	296	0.793 × 10^-9^	0.489 × 10^-9^	1.4 × 10^-9^

*H. cydno-H. heurippa*	μ_*Co*_	1517	2.88 × 10^-9^	1.7 × 10^-9^	7.4 × 10^-9^
	μ_*Tpi*_	416	0.683 × 10^-9^	0.372 × 10^-9^	1.6 × 10^-9^
	μ_*Dll*_	412	1.5 × 10^-9^	0.712 × 10^-9^	4.1 × 10^-9^
	μ_*inv*_	250	1.8 × 10^-9^	0.865 × 10^-9^	2.8 × 10^-9^
	μ_*sd*_	308	0.61 × 10^-9^	0.29 × 10^-9^	1.7 × 10^-9^
	μ_*W*_	296	1.3 × 10^-9^	0.702 × 10^-9^	4 × 10^-9^

*H. melpomene-H. heurippa*	μ_*Co*_	1517	2.88 × 10^-9^	1.7 × 10^-9^	8.1 × 10^-9^
	μ_*Tpi*_	416	0.184 × 10^-9^	0.122 × 10^-9^	0.829 × 10^-9^
	μ_*Dll*_	412	5.4 × 10^-9^	1.5 × 10^-9^	7.1 × 10^-9^
	μ_*inv*_	125	0.583 × 10^-9^	0.215 × 10^-9^	1.2 × 10^-9^
	μ_*sd*_	308	5.5 × 10^-9^	2.7 × 10^-9^	1.09 × 10^-8^
	μ_*W*_	296	1.1 × 10^-9^	0.399 × 10^-9^	3.5 × 10^-9^

μ_*x*: _mutation rate per base pair per generation for each locus.

In the *H. cydno *– *H. heurippa *comparison, *inv *had recombination (p = 0.015) with the most significant breakpoint found between sequences H3-1 and C8-2 between 52^nd ^and 53^rd ^sites. A non recombining block of 250 bp preceding the sites involved in the recombination was selected, in the 5' to 3' direction (Table 4). In this comparison, *sd *also showed significant recombination between sequences H1-1 and H4-1 with the most significant breakpoint at the 16^th ^and 17^th ^sites (p = 0.0001). For this locus, a 308 bp region (5' to 3') lacking recombination was included in further analysis (Table 4). In the *H. melpomene *– *H. heurippa *comparison, the only locus with recombination was *inv *in which, the exchange was between the sequences H7-2 and M7-1, with the most significant breakpoint between 12^th ^and 13^th ^sites (p = 0.005). A 125 bp 5' to 3' region after the sites involved in recombination was selected for the IM analysis (Table 4).

### History of divergence between *H. melpomene and H. cydno*

Isolation-Migration model [38] was used to infer the population history of *H. melpomene melpomene *and *H. cydno cordula*. The IM program uses a coalescent model to estimate effective population size, time since divergence, and ongoing migration parameters from multilocus sequence data sampled from two sister species. Here, *H. cydno cordula *was estimated to have a two-fold greater population size compared to *H. melpomene melpomene *(Table 5). This result is consistent with other studies involving different *H. cydno *and *H. melpomene *geographical races [37,39]. The ancestral population size was 1.23 × 10^6 ^(0.76 × 10^6^-1.98 × 10^6^) individuals and the speciation event probably took place around 2 million years ago, similar to a previous estimate using a different dataset [37]. However, because of the weak scalar estimation for this parameter, this estimated time is a very approximate value (Table 5).

**Table 5:** Genealogical parameters estimated under the IM model

Species comparison	θ_1_	θ_2_	t	mtDNA		*Tpi*		*inv*		*Dll*		*w*		*sd*	
				a	b	a	b	a	B	a	b	a	b	a	b
*H. melpomene*	0.017	0.041	0.01	1.32 × 10^-6^	~0	~0	~0	~0	~0	~0	~0	0.61 × 10^-6^	~0	~0	~0
	0.022	0.028	0.005	0.17 × 10^-6^	~0	~0	~0	~0	~0	~0	~0	~0	~0	~0	~0
*H. cydno*	0.041	0.12	0.04	1.68 × 10^-5^	8.68 × 10^-5^	5.74 × 10^-5^	2.24 × 10^-4^‡	7.99 × 10^-5^	2.31 × 10^-5^	1.32 × 10^-4^‡	3.45 × 10^-4^‡	8.7 × 10^-5^	2.7 × 10^-4^‡	5.44 × 10^-5^	1.68 × 10^-4^‡
	1,121,086	2,042,530	1,971,860‡												
	0.041	0.0059		~0	~0	~0	~0	2.64 × 10^-5^	2.96 × 10^-6^	4.12 × 10^-6^	~0	1.05 × 10^-5^	0.016 × 10^-6^	2.25 × 10^-6^	2.21 × 10^-5^
*H. cydno*	0.028	0.0053		~0	~0	~0	~0	5.88 × 10^-6^	0.01 × 10^-6^	1.70 × 10^-6^	~0	1.89 × 10^-6^	~0	0.90 × 10^-6^	4.37 × 10^-6^
*H. heurippa*	0.12	0.011	----	7.17 × 10^-5^	3.59 × 10^-6^	1.29 × 10^-4^‡	9.24 × 10^-6^	3.47 × 10^-4^‡	2.24 × 10^-5^	3.22 × 10^-4^‡	2.12 × 10^-5^	3.94 × 10^-4^‡	4.12 × 10^-5^	2.38 × 10^-4^‡	4.32 × 10^-5^
	1,903,673	274,516													
	0.0060	0.014		~0	~0	~0	~0	4.48 × 10^-6^	0.07 × 10^-6^	0.027 × 10^-6^	2.48 × 10^-5^	1.01 × 10^-5^	0.029 × 10^-6^	1.36 × 10^-6^	5.67 × 10^-5^
*H. heurippa*	0.0056	0.007	----	~0	~0	~0	~0	0.03 × 10^-6^	~0	~0	5.45 × 10^-6^	0.018 × 10^-6^	0.015 × 10^-6^	0.011 × 10^-6^	4.7 × 10^-6^
*H. melpomene*	0.010	0.033		3.15 × 10^-6^	4.71 × 10^-6^	7.64 × 10^-6^	1.09 × 10^-5^	4.42 × 10^-5^	8.71 × 10^-5^	7.15 × 10^-6^	7.97 × 10^-5^	3.22 × 10^-5^	8.81 × 10^-5^	1.70 × 10^-5^	1.34 × 10^-4^‡
	295,349	701,033													

For each cell the first value is the parameter estimate, second is the lower limit and third is upper limit at 95% level. ---- indicates that no reliable ML estimate was obtained for a parameter, ‡ unreliable estimate or limit due to flat or incomplete posterior probability distribution sampled and ~0 effectively zero, although the lowest 'bin' does not actually include zero (i.e. low gene flow is probable). a, refers to the forward migration rate (per generation per locus) of haplotypes from population 2 into population 1. b, refers to the forward migration rate (per generation per locus) of haplotypes from population 1 into population 2.

Two loci (mtDNA and *w*) showed evidence of gene flow. In both cases it was asymmetric (Table 5). All remaining genes had estimates asymptotically near to zero in both directions (Table 5). The estimated migration rate from *H. c. cordula *to *H. m. melpomene *(m_1 _= 1.32 × 10^-6 ^per generation per locus) was high at mtDNA, as was expected from the pattern of shared variation seen in the mtDNA tree. Gene flow estimated in the other direction was nearly zero (Table 5). The *w *locus had a 0.61 × 10^-6 ^per generation per locus migration rate in the same direction, a result that is consistent with the presence of the M6-1 *H. melpomene *haplotype in the *H. cydno *allele cluster.

### Isolation-Migration model including *H. heurippa*

The IM model was then used to compare *H. heurippa *with each of the parental taxa. The estimated *H. heurippa *population size was similar (0.27 – 0.30 × 10^6^) in each comparison and smaller than those estimated for the other species (Table 5). Unlike the comparison between *H. m. melpomene *and *H. c. cordula*, both comparisons involving *H. heurippa *failed to yield precise estimates of either the ancestral population size or the divergence time. The likelihood surfaces obtained for these parameters were either flat or rising over the parameter range investigated.

More importantly here, was the incidence of gene flow observed at several loci between *H. heurippa *and the other species, the only exceptions being mtDNA and *Tpi*, where the migration rate was not significantly different to zero in both directions (Table 5). At all other loci migration parameters were significantly greater than zero in at least one direction, with evidence for gene flow both from parental species into *H. heurippa *and vice-versa (Table 5).

## Discussion

### Genealogical pattern and introgression

*Heliconius heurippa *was initially identified as a putative hybrid species based on its intermediate colour pattern, which shows a striking similarity to phenotypes produced after just a few generations of hybridization between *H. c. cordula *and *H. m. melpomene *[26]. The sequence data presented here provides independent evidence that hybridization has played an important, and ongoing, role in the evolution of the *H. heurippa *genome. All four autosomal loci showed a pattern in which *H. heurippa *shares similar alleles with both *H. m. melpomene *and *H. c. cordula*. At three of these loci (*inv, w *and *sd)*, *H. heurippa *was most closely related to *H. c. cordula*, while at the fourth (*Dll*) it was closer to *H. m. melpomene*. Nonetheless, our analysis suggests that the pattern of shared alleles between *H. heurippa *and its relatives is mainly due to introgression in the fairly recent past. The results are consistent with historical gene exchange playing an important role in the origin of *H. heurippa*, but do not provide evidence for a 'mosaic' hybrid genome as has been demonstrated in other examples of hybrid speciation.

### Gene flow between *H. c. cordula *and *H. m. melpomene*

The comparison of *H. c. cordula *and *H. m. melpomene *under the IM model indicates ongoing introgressive hybridization at two loci (Table 5). In particular, there are very closely related mtDNA haplotypes shared between the species (gene flow from *H. c. cordula *to *H. m. melpomene*, m_1 _= 1.32 × 10^-6 ^per generation per locus; Figure 1) and asymmetric moderate gene flow in *w *(m_1 _= 0.61 × 10^-6 ^per generation per locus, gene flow from *H. c. cordula *to *H. m. melpomene *and 0 in the other direction). These species are known to hybridize in the wild and previously, shared alleles have been observed at another autosomal locus, *Mpi *for which a symmetrical migration rate of 1.54 ×10^-6 ^per generation per locus was estimated in Panamanian populations [37,40]. Furthermore, substantial shared DNA sequence variation at 16 loci was observed in *H. cydno *and *H. melpomene *from Costa Rica [39].

Notably, shared mtDNA variation between *H. cydno *and *H. melpomene *in eastern Colombia (Figure 1) suggests recent introgression. The shared mtDNA haplotypes may have been retained as an ancient polymorphism since the speciation of *H. melpomene *and *H. cydno*, but this seems unlikely given the evolutionary distance between the species (1.5–2% divergence between the two mtDNA clades). Female hybrid sterility following Haldane's rule [41] would be expected to prevent introgression of mtDNA, although limited fertility of female hybrids has been documented and would provide a route for infrequent introgression [41].

### The history of *H. heurippa*

Pairwise IM analyses of *H. heurippa *and either one of the other taxa, suggest a small species population size (Table 5). The absence of consistent negative Tajima's D across loci (Table 2) indicates a historically small constant size instead of a short bottleneck. No reliable estimates for divergence time and ancestral population size were obtained in either comparison. Two loci mtDNA and *Tpi *did not show gene flow for any pair evaluated that involves *H. heurippa*. The remaining loci showed recent gene interchange among *H. heurippa *and the two other species. There is no hybrid sterility between *H. cydno *and *H. heurippa*, and insectary mate choice experiments produce frequent matings in one direction (between *H. heurippa *males and *H. cydno *females). It seems likely that there is a hybrid zone between these species somewhere in the Andes between Villavicencio (Colombia) and San Cristobal (Venezuela; Linares Pers. Obs.), which would explain the observed levels of gene flow.

In the *H. melpomene *– *H. heurippa *species pair, *inv *(64) and *w *(348.2) in particular showed high rates of gene flow from *H. melpomene *to *H. heurippa*. These two species are broadly sympatric, which might facilitate hybridization. Nonetheless, although hybrid females are sterile in one direction of cross, female offspring of backcrossed males segregate for sterility which should allow some gene flow [24].

In a broad sense, our Isolation-Migration analysis suggests that different parts of the *H. heurippa *genome are subject to very different levels of gene flow with the two sister taxa. We also carried out linkage disequilibrium tests for introgression [42], which should be sensitive to very recent introgression events, but these were not significant for any of the loci, suggesting that gene flow is sufficiently ancient for linkage disequilibrium to have been lost. There are a number of possible explanations for such interlocus variation. One is that some of the loci sequenced for this study are either themselves subject to natural selection or else are linked to such regions. Selection does seem plausible as we know that there is a strong correlation between the *Tpi *locus and hybrid sterility in interracial *H. melpomene*, *H. cydno *x *H. melpomene *and *H. melpomene *x *H. heurippa *crosses [24,41,43]. If *Tpi *were associated with genes causing hybrid sterility, this might explain the clear lack of gene flow among the three species at this locus. This is also consistent with the fact that a disproportionate number of species differences, including morphology, physiology, oviposition preference, mate selection and pheromones, map to the Z chromosome in other Lepidoptera species [44].

At the other extreme, *sd *shows far more allelic mixing between species than the other loci studied. A similar pattern in the cydno-melpomene group races from Panama and Costa Rica has been observed at other loci [37,39]. This has led to suggestion that balancing selection could be maintaining diversity and perhaps promoting introgressive hybridization [37,39,40]. It is possible that this process is occurring at the *sd *haplotypes obtained here. There is currently no evidence for the role of *sd*, or any of the other loci studied here playing a role in the evolution of colour pattern, so it is unclear what the selection pressures on this locus might be.

In addition, analysis of protein coding sequence did not provide any direct evidence for positive or balancing selection on any of the loci, although the power of this analysis was limited by the fact that most of our sequence data is for non-coding regions (analysis using CODEML, data not shown, [45]). The genealogical pattern and IM analysis do suggest that there is both ancestral variation and recent gene flow at the *sd *locus (Table 5).

Overall, the species relationships are consistent with the *H. heurippa *genome containing a greater contribution from *H. cydno *than *H. melpomene*. The *H. heurippa *mtDNA haplotypes fall within an *H. cydno *clade, and at three of the five nuclear loci F_ST _values show *H. heurippa *closer to *H. cydno *(Table 3). This is consistent with what is known about the three species. *H. heurippa *is a geographic replacement of *H. cydno *that flies in similar habitats. Where *H. cydno *flies sympatrically with *H. melpomene*, the former is associated more with closed canopy forest and tends to be found at higher altitudes [46], both characteristics also observed in *H. heurippa *populations in eastern Colombia (Linares and Jiggins Pers. Obs.). Furthermore, laboratory reconstruction of the *H. heurippa *colour pattern from *H. c. cordula *and *H. m. melpomene *involves backcrossing F_1 _male hybrids to *H. cydno*, with a correspondingly greater contribution from the *H. cydno *genome. Finally, patterns of hybrid sterility show that *H. heurippa *is more compatible with *H. cydno *[24,26]. Nonetheless, the *Tpi *genealogy contrasts with this general pattern, in that *H. heurippa *is similarly differentiated from both of the other species, suggesting a more contemporaneous divergence of all three species. This contrasts with the other genetic and ecological evidence placing *H. heurippa *as a closer relative of *H. cydno*, and might indicate an even more complex evolutionary history.

In summary, the data clearly imply that introgressive hybridization has occurred between the three species, but this does not distinguish between two alternative scenarios for the origin of *H. heurippa*. First, a branching speciation scenario with ongoing gene flow, and second a hybrid founding speciation scenario. Thus, these data alone cannot be taken as providing strong evidence in support of hybrid speciation. Indeed, the observed pattern of allelic variation contrasts markedly with the pattern seen in hybrid *Helianthus *sunflowers [7,47]. The latter species show genomes consisting of genetic "blocks" derived from one or the other of the parental species. These would be seen in our data as a clustering of the hybrid species entirely within one or the other parental species for each locus. However, this is not the general pattern seen in *H. heurippa*, where we observe allelic variation shared with both parental species at several loci. Intuitively, the latter pattern seems more consistent with high ongoing rates of introgression at many loci, and species differences maintained by selection at the remaining loci. This contrast is perhaps unsurprising given that the hybrid sunflowers are largely allopatric to their progenitors, while *H. heurippa *is still able to exchange genes with both of its parental species.

Superficially, the data seems to support the hybrid speciation hypothesis, with F_ST _analyses showing *H. heurippa *more related to *H. cydno *at some loci and more related to *H. melpomene *at others. This is the kind of pattern that has been used previously to argue the case for hybrid speciation in other groups [2,48,49]. Furthermore, there are clearly alleles in *H. heurippa *that could be considered diagnostic for one or other of the parental species at different loci, similar to the 'private alleles' found in the 'Lonicera fly' and 'alpine *Lycaeides*' that were apparently derived from one or other putative parent [50,51]. Thus, our data are similar in several aspects to previous examples that have been proposed as good cases for hybrid speciation. Nevertheless, we would argue that such evidence needs to be combined with analysis of traits involved in causing reproductive isolation in order to argue convincingly that hybridization played a causative role in speciation.

## Conclusion

Our results highlight the difficulty of clearly proving the case for hybrid speciation. If hybrid speciation is important, it must often occur in taxa with significant rates of introgressive hybridization, such that where shared variation is observed the alternative hypotheses of hybrid founding versus introgressive hybridization need to be rigorously tested. The evidence for the role of hybridization in the speciation of *H. heurippa *comes primarily from crossing and mate choice experiments that have addressed the origin of its colour pattern, and the role of that pattern in reproductive isolation [26]. That colour pattern is controlled by a small number of genes of major effect, so the hybrid speciation hypothesis does not make specific predictions regarding the rest of the genome. Here we have clearly shown that *H. heurippa *does not have a hybrid genome that is comparable to the sunflower *Helianthus anomalus*, with blocks of genes derived from one or other parent. Instead, the genome resembles that of other groups of closely related taxa in which hybridization is frequent, such as *Anopheles gambie *[52,53] and the *Drosophila pseudoobscura *group [38,42,54].

Discordant patterns are found at different markers such that particular alleles cannot be considered 'diagnostic' of a particular species. Thus, in this case hybrid speciation has resulted from the origin of a novel trait with a key role in speciation. Thus, in the case of *H. heurippa*, sequence analysis of the genes controlling the different pattern elements will be needed to uncover the genealogical history of the original speciation event.

## Methods

### Specimen collection, PCR and sequencing

Butterflies were collected in Colombia and Venezuela (see additional file 1: Specimen collection list), wings removed and stored in glassine envelopes. The bodies were preserved with 100% EtOH in the Universidad de Los Andes (M code) and in DMSO in the Jiggins collection (stri-b code) for *H. heurippa *(n = 11), *H. m. melpomene *(n = 8) and *H. c. cordula *(n = 9) from the eastern Colombian and Venezuelan Andes (see additional file 1: Specimen collection list). Cytochrome oxidase subunit 1 (CoI)/tRNA^LEU^/cytochrome oxidase subunit 2 (CoII) region and the Z-linked locus, Triose-phosphate isomerase (*Tpi*), were amplified and sequenced from individual genomic DNA using PCR primers and conditions described previously [40]. All nuclear gene products were cloned before sequencing using pGEM-T Easy Vector System (Promega). For each individual 3–5 clones were sequenced to identify distinct alleles. Previously Distal-less (*Dll*) and invected (*inv*) sequences obtained by CS [26,29] and the newest white (*w) *and scalloped (*sd*) sequences were amplified from individual genomic DNA using PCR primers and conditions already described [30]. Sequences included in the analysis were generally represented by at least two identical clones. All loci sequences were deposited in GenBank under accessions [DQ674383-DQ674451, DQ445385-DQ445415 and DQ445416-DQ445457].

### Phylogenetic analysis

Bayesian and parsimony phylogenetic trees were reconstructed as described previously for mtDNA data [40]. Recombination violates the assumption of a bifurcating genealogy [54], so for nuclear loci we constructed haplotype networks that take into account the presence of persistent ancestral nodes, multifurcations and reticulation. The presence of loops in these networks might reflect recombination events [55]. Networks for nuclear loci were constructed with statistical parsimony in TCS v 1.21 [56], considering gaps as missing data and adjusting the parsimony limit to the respective data set.

### Population genetic analysis

For each species, the per site population mutation rate Θ_W_[57] with 95% confidence intervals was estimated with DnaSP v4.10.3 [58]. Deviation from a neutral model, and hence the effectiveness of Θ_W _in reflecting the effective population size (Ne) was tested by estimating Tajima's D [59]. Significant departure of Tajima's D from zero was evaluated both assuming recombination and without recombination. Both tests were carried out because presence of recombinant sites in the nuclear genes leads to a loss of power to reject neutrality [60]. For between species comparisons, the program SITES [61] was used to estimate net divergence between species [62] and the number of shared polymorphisms and fixed differences. Genetic differentiation between pairs of populations was measured using Wright's F_ST _[63] adapted for DNA sequence data [64,65] and estimated using DnaSP v4.10.3 [58]. Statistical significance was obtained by bootstrapping i.e. randomly sampling with replacement the values of within population diversity, π_S_, and the values of the between population divergence, π_B_, 10000 times and recalculating F_ST _for each replicate using SEQUENCER v6.1.0 [66].

In order to estimate the role of gene flow in shaping the pattern of shared alleles between species, the Isolation-Migration (IM) bayesian model was used [38]. This model considers a scenario where a population gives rise to two populations, after which there may be gene exchange between the two new populations [38]. The program relies on a metropolis-coupled Monte Carlo Markov chain (MCMC) genealogical sampling for Bayesian estimation of six major demographical parameters: recent population sizes, ancestral population size, time at which the ancestral population bifurcated and two or more migration rates according to whether gene flow is evaluated at the population or gene level [38]. The genealogical coalescence parameter estimation follows the classical Kingman theory in which recent alleles of two daughter populations will be traced to an ancestral pool in a Wright-Fisher fashion [38]. In this context, the genetic processes of mutation and drift occurs on a time scale of generations. Because of this, the parameters must be scaled based on a known mutation or drift rate.

We carried out analyses on three modified couplet datasets for each species pair: (i) *H. melpomene-H. cydno *(ii) *H. melpomene-H. heurippa *and (iii) *H. cydno-H. heurippa*. For the former comparison we expected that IM model fits well to the data. However, in the couplet data sets involving *H. heurippa*, IM could overestimate the divergence time and underestimate the migration rates between this species and either parent species. This is a consequence of the presence within *H. heurippa *of divergent alleles that relate it with the species not included in the IM comparison. Nonetheless, the coalescence process in the IM simulations is able to recover information about the ancestry of shared and divergent alleles between *H. heurippa *and any of the other two species and provided an approximation of the magnitude of gene flow.

Additionally, in concordance with the typical assumptions and limitations of the IM algorithm (i.e. no gap polymorphism, no recombination within loci), we looked for regions in our sequences that met those conditions. For each alignment, we obtained a dataset that was as complete as possible after deleting highly polymorphic indel regions. Over these data sets, evidence for recombination was evaluated by the Hudson four gamete test implemented in SITES [67]. However, this test assumes an infinite sites mutation model that is not realistic for our data due to observation of repeated changes at the same site under the HKY model [68]. Hence, recombination was also tested using a model-neutral test based on a bootstrapped correlation of linkage disequilibrium (*R*2) with physical distance and with a maximum chi-square test [69,70]. Both analyses were used to subsample the indel-free files in order to remove clearly recombinant regions by searching from the 5' region of each locus until a probable recombinant pattern emerge [37]. The same procedure was made from the 3' region. From 5' and 3' analysis, we selected a maximum block without recombination [37]. Effective population size scalars were assigned to each locus relative to autosomal inheritance. Specifically, the values were set at 0.25 for CO, 0.75 for *Tpi *and 1 for the other loci. Individual species population sizes and ancestral population size (θ = 4Nμ), divergence time, relative mutation rate μ and per locus directional migration rates (*m*) were estimated with all loci. Demographic values were obtained through a molecular clock scalar calibration to obtain parameters per base pair and per generation, taking as reference Brower's 2.3% divergence in mtDNA per million years estimated for insects [71] and with an assumption of four generations per year (Table 4). Migration rates (*m*_1 _y *m*_2_) were allowed to vary between loci and between species (i.e. asymmetrical gene flow, option -j6). After search for parameter range using preliminary runs, each pairwise comparison were run for at least 30 million steps from a 200,000 burn-in period under the HKY model [68] with 5 chains per set, with linear heating increment *h *of 0.033.

## Authors' contributions

CS carried out laboratory work and genetic analyses. CDJ helped in data analysis and, with CS, drafted the manuscript. JET helped with statistical analysis. MRK carried out nuclear primer designing and provided reference sequences. ML participated in specimen collection and in the design of the study. All authors participated in the conception of the study and approval of the final manuscript.

## Supplementary Material

Additional file 1 **Specimen collection list**. Populations sampled with information about the genes sequenced for each individual. Click here for file
