# Supplementary material for: Gene flow and the genealogical history of Heliconius heurippa
Source: BMC Evol Biol. 2008 May 2;8:132. doi: 10.1186/1471-2148-8-132 (PMC2391162; doi:10.1186/1471-2148-8-132)
Supplement: Additional file 1 — Specimen collection list. Populations sampled with information about the genes sequenced for each individual. [file 1471-2148-8-132-S1.pdf]

Figure code	Identification number	species	Locality	CO[a]	*Tpi*[a]	*Dll*	*inv*	*w*	*sd*	Collection date
C1	M187	*H. c. cordula*	San Cristobal, Merida , Venezuela 7°47’35’’ N, 72°11’44’’W	*	*	√	√	√	√	16/10/02
C2	M105	*H. c. cordula*	San Cristobal, Merida , Venezuela 7°47’35’’ N, 72°11’44’’W	*	-	√	√	√	-	13/06/02
C3	M104	*H. c. cordula*	San Cristobal, Merida , Venezuela 7°47’35’’ N, 72°11’44’’W	*	*	√	-	√	√	13/06/02
C4	M101	*H. c. cordula*	San Cristobal, Merida , Venezuela 7°47’35’’ N, 72°11’44’’W	*	*	√	√	√	√	13/06/02
C5	M182	*H. c. cordula*	San Cristobal, Merida , Venezuela 7°47’35’’ N, 72°11’44’’W	*	*	√	-	√	√	16/10/02
C6	M110	*H. c. cordula*	San Cristobal, Merida , Venezuela 7°47’35’’ N, 72°11’44’’W	-	-	√	√	-	-	9/08/02
C7	M111	*H. c. cordula*	San Cristobal, Merida , Venezuela 7°47’35’’ N, 72°11’44’’W	-	-	-	√	-	-	9/08/02
C8	M189	*H. c. cordula*	San Cristobal, Merida , Venezuela 7°47’35’’ N, 72°11’44’’W	-	-	-	√	-	-	9/08/02
C9	M199	*H. c. cordula*	San Cristobal, Merida , Venezuela 7°47’35’’ N, 72°11’44’’W	-	-	-	√	-	-	9/08/02
M1	Stri-b-13	*H. m.melpomene*	Chirajara, Cundinamarca, Co. 4° 12’ 48’’N, 73°47’70’’W	*	*	√	√	√	-	28/02/98
M2	M113	*H. m. melpomene*	Santa Ana, Merida, Venezuela 7° 36’41’’N, 72°18’10’’W	*	*	√	√	√	√	13/06/02
M3	M95	*H. m. melpomene*	Chirajara, Cundinamarca, Co. 4° 12’ 48’’N, 73°47’70’’W	*	*	√	√	√	√	15/07/02
M4	Stri-b-9	*H. m. melpomene*	Chirajara, Cundinamarca, Co. 4° 12’ 48’’N, 73°47’70’’W	*	*	√	√	√	√	28/02/98
M5	Stri-b-11	*H. m. melpomene*	Chirajara, Cundinamarca, Co. 4° 12’ 48’’N, 73°47’70’’W	*	*	√	√	√	-	28/02/98
M6	Stri-b-12	*H. m. melpomene*	Chirajara, Cundinamarca, Co. 4° 12’ 48’’N, 73°47’70’’W	*	*	√	√	√	√	28/02/98
M7	M119	*H. m. melpomene*	Santa Ana, Merida, Venezuela 7° 36’41’’N, 72°18’10’’W	*	*	-	√	-	-	9/08/02
M8	M115	*H. m. melpomene*	Santa Ana, Merida, Venezuela 7° 36’41’’N, 72°18’10’’W	*	*	-	-	-	-	13/06/02
H1	Stri-b-40	*H. heurippa*	Chirajara, Cundinamarca, Co. 4° 12’ 48’’N, 73°47’70’’W	√	√	√	√	√	√	28/02/98

H2	Stri-b-51	*H. heurippa*	Chirajara, Cundinamarca, Co. 4° 12’ 48’’N, 73°47’70’’W	√	√	√	√	√	-	28/02/98
H3	Stri-b-44	*H. heurippa*	Chirajara, Cundinamarca, Co. 4° 12’ 48’’N, 73°47’70’’W	√	√	√	√	√	√	28/02/98
H4	M17	*H. heurippa*	Chirajara, Cundinamarca, Co. 4° 12’ 48’’N, 73°47’70’’W	√	√	√	-	√	√	2/08/02
H5	M12	*H. heurippa*	Chirajara, Cundinamarca, Co. 4° 12’ 48’’N, 73°47’70’’W	√	√	√	√	√	√	2/08/02
H6	M145	*H. heurippa*	Chirajara, Cundinamarca, Co. 4° 12’ 48’’N, 73°47’70’’W	√	-	√	√	-	-	15/07/02
H7	Stri-b-39	*H. heurippa*	Chirajara, Cundinamarca, Co. 4° 12’ 48’’N, 73°47’70’’W	√	√	√	√	√	-	28/02/98
H8	M8	*H. heurippa*	Chirajara, Cundinamarca, Co. 4° 12’ 48’’N, 73°47’70’’W	√	√	√	√	-	-	2/08/02
H9	Stri-b-34	*H. heurippa*	Chirajara, Cundinamarca, Co. 4° 12’ 48’’N, 73°47’70’’W	√	√	-	√	-	-	28/02/98
H10	M141	*H. heurippa*	Chirajara, Cundinamarca, Co. 4° 12’ 48’’N, 73°47’70’’W	√	√	-	√	-	-	15/07/02
H11	M4	*H. heurippa*	Chirajara, Cundinamarca, Co. 4° 12’ 48’’N, 73°47’70’’W	√	√	-	-	-	-	2/08/02

[a] Mitochondrial and sex-linked sequences for *H. c. cordula* (C) and *H. m. melpomene* (M) indicated with * were taken from GenBank. (DQ019244-DQ019246, DQ019250,DQ019251, DQ19234-DQ19239 for *H. c. cordula* and AY548139, DQ019243, DQ019247-DQ019249, DQ019252-DQ019254, DQ019228-DQ019233, DQ019240, AY548151 for *H. m. melpomene*). The following CoI and CoII sequences were also included: *H. c. chioneus* (AF413672, AF413707, AF512978, AF512980, AF512985, AF512989, AF512990-AF512993, AY548130), *H. m. rosina* (AF413673, AF413674, AF512971, AF512972, AF512977, AF512982-AF512984, AF512987), *H. c. weymeri* (AY548114-AY548116), *H. m. mocoa* (AY548118-AY548129, AY548131-AY548138) and *H. hecale* (AF413683).
